# Investigation of Stress Level Among Dentistry Students, General Dentists, and Pediatric Dental Specialists During Performing Pediatric Dentistry in Kerman, Iran, in 2017

**DOI:** 10.2174/1745017901814010631

**Published:** 2018-09-28

**Authors:** Elham Farokh-Gisour, Marjan Hatamvand

**Affiliations:** 1 Pediatric Dentistry Department, Center of Endodontic Research, Kerman University of Medical Sciences, Kerman, Iran; 2 Pediatric Dentistry Department, Kerman University of Medical Sciences, Kerman, Iran

**Keywords:** Stress, Anxiety, Pediatric dentistry, Maxilla, Mandible, General dentists

## Abstract

**Background & Aim:**

Dentists are exposed to stress and tension as they have a close contact with the patients. The increase in stress may affect the dentists’ performance and can be a major threat to the physical and mental health of the patients. Pediatric dentistry requires experience, without which the amateur dentists and students feel lack of self-confidence, and consequently be unable to deal with problematic patients. There is no study investigating stress during pediatric dentistry among the dentistry students as well as general and pediatric dentists in Iran. Regarding the importance ofthis subject, we aimed to evaluate the stress level among the aforementioned three dental service providers during pediatric dentistry.

**Materials & Methods:**

This study was conducted on 300 dentistry students, general dentists, and pediatric dental specialists in Kerman, Iran. The data were collected using a questionnaire entailing demographic information and therapeutic practices of pediatric dentistry. After checking the participant’s comments, the data were analyzed using *SPSS* version 16 (IBM, Texas, USA).

**Results:**

According to the results, the mean age of the participants was 28±5 years. Out of the 300 participants, 113 (37.7%) and 178 (59.3%) cases were male and female, respectively, and 9 (3%) participants did not fill out this part. Furthermore, 108 (36%), 173 (57.7%), 6 (2%), and 10 (3.3%) subjects were general students, general dentists, residents, and pediatric residents, respectively. Anesthesia injection in the mandible for an anxious child and amalgam restoration in the mandible led to the highest and lowest stress levels in the participants, respectively. Furthermore, the mean stress levels were significantly different between the practices performed in the maxilla and mandible (*P*<0.001). In addition, the females showed a significantly higher level of stress, compared to the males (*P*<0.001). The specialists had significantly lower stress than the dentistry students and general dentists (*P*<0.001).

**Conclusion:**

As the findings indicated, anesthetic injection to a nervous infant was the most stressful practice in pediatric dentistry. The results also showed that the pediatric dental specialists had lower stress level, compared to the students and general dentists.

## 1 INTRODUCTION

Dentistry is considered a stressful occupation [1-3]. as the dentists have close contact with the patients [4, 5]. The increase in stress may result in affecting the performance of the dentists, which can be a major threat to the physical and mental health of the patients [6]. Pediatric dentistry is a branch of dentistry that is associated with the provision of dental services for the children and adolescents under the age of 18 years [7-9].

In Iran, the general dentistry students are required to pass the theoretical and practical courses on the pediatric field over a short period. Accordingly, the pediatric dentists have to complete all pediatric dentistry courses in three years [10]. Despite the training presented to the dentists during the general and expertise periods, dental practices exert great stress on the clinicians due to their high importance and sensitivity [11-13]. There are a large number of dentists who do not enjoy working with children.

The pediatric dentists are exposed to children's crying, jerking, anger, and a variety of other avoidance behaviors. These conditions cause dentists to be irritable or make them nervous since they have to spend energy to stop them and adapt to such behaviors [14]. In a study conducted by Chipchase in 2017, the dentists with higher anxiety and stress levels were more likely to report changes in clinical methods [15].

One of the aspects of pediatric dentistry is experience, without which the young students and dentists feel lack of self-confidence, and consequently be unable to deal with problematic patients [16-18]. The previous studies investigating the clinicians’ stress levels during dental work have reported a relatively high level of stress in some dental practices [19, 20]. Therefore, it seems that dentistry practices are stressful for both patients and dentists.

To the best of our knowledge, there is no study investigating this domain, especially stress during pediatric dentistry among the dentistry students as well as general and pediatric dentists in Iran. Regarding this and considering the importance of this subject, we aimed to evaluate stress level among the aforementioned three dental service providers in pediatric dentistry. The findings of this study could be helpful in revealing the educational needs of the general and specialized students regarding the implementation of stress control programs. Moreover, this study is a good attempt to find solutions to reduce the stress of general and specialized dentists.

## 2 MATERIALS AND METHODS

This descriptive cross-sectional study was conducted on general dentistry students, who completed the pediatric dental practical courses, general dentists, and pediatric dentists in Kerman, Iran, in 2017. Sampling was performed by obtaining the name list of all general and pediatric dentists working in Kerman from the medical system and dentistry faculty and referring to their workplace. In addition, all dental students who had passed at least two semesters of pediatric courses were included in the study in the second semester of 2016-2017. After obtaining approval of the Ethics Committee of Kerman University of Medical Sciences, Kerman, Iran, the questionnaires were submitted to the subjects, and they were assured about the confidentiality terms.

### 2.1 Data Collection Instrument

The data were collected using a questionnaire, which was previously employed by Davidovich *et al*., [21]. Based on the dental practices in Kerman, a number of items were changed, and one item was removed. The dependent variable in the present study was the stress level during pediatric dentistry, which was rated on an 11-point Likert scale (ranging within 0=no stress to 11=severe stress).

After the evaluation of the subjects' views, the questionnaires were coded and then analyzed in SPSS software (version 16, IBM, Texas, USA). Each item was assigned the score value of one; accordingly, the total score was estimated for comparative purposes. After data collection, the stress scores were divided into four groups. In this regard, the stress scores at the 0-10^th^, 11-50^th^, 51-90^th^, and 91-100^th^ percentiles were considered as showing low, moderate, high, and severely high-stress levels, respectively.

### 2.2 Sample Size

The sample size included all dental students who completed 2-4 pediatric courses in the second semester of the academic year of 2016-2017, as well as all general and pediatric dentists working in Kerman. Accordingly, those who were willing to participate in the study entered into the research process.

### 2.3 Data Analysis

Data analysis was performed using descriptive statistics (**e.g**., mean and standard deviation). In addition, a t-test was conducted to compare the stress levels in terms of gender as well as maxillary and mandibular practices. The comparison of the three groups (*i.e*., students, as well as general and expert dentists) regarding the stress levels of each practice performed in the maxilla and mandible was accomplished through using two-way ANOVA test.

### 2.4 Ethical Considerations

To ensure the confidentiality of the data, name or label was specified in all questionnaires. Furthermore, the participants were informed about the objective of the study and applicability of the findings. Additionally, they completed the questionnaire willingly, and the participation in the study was completely voluntarily. The questionnaire was approved with the code number of ethics 1354. 1396. IR.KMU.REC.

The validity of the questionnaire was investigated with five pediatric dentist students, rendering a Cronbach's alpha coefficient of 0.76. The reliability of this instrument was also confirmed by presenting it to 10 students and dentists within two weeks, resulting in a Cronbach’s alpha coefficient of 0.8.

## 3 RESULTS

The mean age of all participants was 28±5 years. Out of the 300 participants, 113 (37.7%) and 178 (59.3%) cases were male and female, respectively, and 9 (3%) participants did not fill out this part. Furthermore, 108 (36%), 173 (57.7%), 6 (2%), and 10 (3.3%) cases were general students, general dentist, residents, and pediatric residents, respectively. However, 3 (1%) participants did not complete this section (Table **1**).

The mean length of employment was 3±4.2 years among the participants. As the results demonstrated, anesthesia injection in the mandible for an anxious child led to the highest stress among the participants (mean: 4.17 ± 1.2). On the other hand, cavity preparation for the amalgam restoration in mandible led to the lowest stress level (mean: 1.11 ± 0.31). There was a significant difference between the practices performed in the maxilla and mandible in terms of the mean stress level (*P*<0.001).

**Fig. (1):**
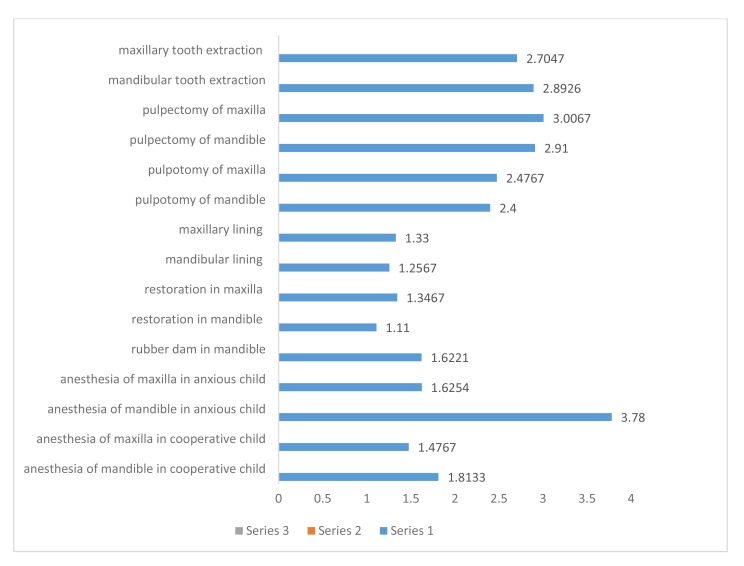
Mean of stress level among general dentistry students, postgraduate students and pedodontists.

However, no significant difference was observed between the two studied groups in terms of placing the rubber dam (*P*=0.9). The females showed significantly higher stress, compared to the males (*P*<0.001). Nonetheless, although females’ stress was higher, no significant difference was observed between the males and females regarding the insertion of the rubber dam and lower mandible restoration (*P*=0.4 and *P*=0.15, respectively). Additionally, the dental specialists showed a significantly lower stress level than the pedodontics postgraduate students and general dentists (*P*<0.001) (Table **2** and Fig. **1**).

## 4 DISCUSSION

The present study was an attempt to determine the stress levels among the dentistry students, general dentists, and pediatric dentists during the pediatric dentistry operations in Iran. According to anesthesia injection for the anxious child was the most stressful part of pediatric dental occupation, which can be explained by the special anatomy of the mandible for injection, and the lack of child's cooperation in opening the mouth completely due to fear and anxiety, which is essential for this type of injection.

These findings are similar to those obtained by Davidovich *et al*, (2015) [21], who showed that the most possibly stressful practice for the general and specialized dentists was the anesthesia injection for an anxious child. These findings are in line with the results obtained by Aishwarya *et al*, (2008), reporting that anesthesia in the pediatric patients was the most stressful dentistry work in 43% of the students [22].

In this study, the least stressful practice was the preparation of the cavity for mandibular amalgam restoration, which could be due to having a direct vision and easier access to mandible rather than the maxilla. However, Roneeberg *et al*, (2015) reported the restoration of the children aged 3-5 years as the most rigorous work of dentistry without specifying the exact type of operation [14].

This can be due to the difficulty of behavioral control in the children under the age of 5 years. The findings of this study demonstrated no significant difference between the practices performed in the mandible and maxilla in terms of the mean stress level during the placement of the rubber dam. This finding can be due to the lack of training or use of rubber dam at the department. In addition, the rubber dam is not usually used in the general dentists’ offices [23].

The results of this study revealed that cutting the crown of the maxillary teeth was more stressful than performing such a practice in the mandible. This can be due to the high visibility and indirect access to maxilla [24]. It was also shown that the stress level during root canal treatment (**i.e**, pulpotomy and pulpectomy) was much higher in the maxilla rather than that in the mandible due to the visibility and indirect accessibility of the maxilla as well as the incomplete opening of mouth by the child.

Furthermore, the presence of zygomatic appendage eliminates the possibility of performing a good anesthetic action for the second molar teeth in the posterior maxilla. On the other hand, the risks causing aspiration problems, such a file drops into the child's throat, make the access to the root canal of the maxillary teeth more difficult. In this study, the stress induced during the maxillary dental extraction was higher than that in the mandibular teeth extraction due to lower access. In addition, the maxillary molars have three roots, which increase their chance of breaking [25]. Moreover, the patient's position during the extraction of the maxillary teeth increases the risk of tooth falling in the patient's throat [26, 27].

In all of the studied practices, the females showed a higher level of stress as compared to the males, except for the insertion of the rubber dam and cutting the crowns in the mandible. These findings are in alignment with those obtained by Gambetta Tessini *et al*, (2013) reported that women are more responsive, sympathetic, and supportive toward the patients, which causes them to show higher stress [28]. These results clearly confirm that the female dentists induce a negative effect on the patients due to their high level of emotional feelings and more subtle mood, especially when they feel patients’ pain or are fearful of dental practices. Accordingly, this causes disturbances of mind and stress and exerts stress on the female dentists.

Myers and Myers (2004) revealed that the patient's stress was significantly correlated with physician's stress and that increased stress in the patients led to elevate stress in the dentists [18]. This issue can be attributed to the fact that in Iran, the female general dentists mostly visit children. Based on the findings of the present study, the specialized dentists had a lower level of stress, compared to the students and general dentists, who are in line with the results reported by Boran *et al*, (2011) [7]. These findings may be due to the higher professional experience of the specialists, compared to that of the students and general dentists. Similarly, Roneeberg *et al*, (2015) reported stress as an important factor among 60% of the cases with less than 10 years of work experience. However, this amount was reduced to 40% for those who had an occupational experience of over 10 years [14].

The comparison of stress among the three groups revealed that the students, general dentists, and specialists had stress levels of medium to severe, moderate, and mild, respectively. This finding indicates that dentistry can be classified under stressful occupations. The specialized dentists have been reported to experience a mild level of stress, compared to others due to having higher work experience and taking more specialized courses [29-31].

The aim of this study was to enhance general knowledge about the stress of pediatric dentists in Kerman province. The findings were indicative of a high level of occupational stress among these dental care providers. The results of the present study can be a starting point for the management of the stress and implementation of training courses for the fields associated with children. Further studies are recommended to be conducted at wider levels to obtain comprehensive information in this regard.

## CONCLUSION

As the findings of the present study indicated, injection of anesthesia to the anxious children was the most stressful practice in pediatric dentistry. Furthermore, the specialists had a lower level of stress, compared to the students and general dentists. According to our findings, pediatric dentistry is a stressful job, which requires the ability to control emotions and stress.

## Figures and Tables

**Table 1:** Demographic data of the participants.

**Variable**		
**Gender**	Male	113 (37.7%)
	Female	178 (59.3%)
**Education level**	Student	108 (36%)
	General dentist	173 (57.7%)
	Resident	6 (2%)
	Pediatric dentist	10 (3.3%)

**Table 2:** Mean stress of different dental practices and their relationship with the practice in the maxilla and mandible.

**Variable**	**Stress based on Jaw**			**Stress based on Gender**		
	Mean	SD	*P*-value	Male (SD)	Female (SD)	*P*-value
Anesthesia of mandible in a cooperative child	1.8	1.6	0.0001	1.1 (1.3)	2.2 (1.7)	0.0001
Anesthesia of maxilla in a cooperative child	1.5	1.4		0.8 (1)	1.8 (1.4)	0.0001
Anesthesia of mandible in an anxious child	4.1	2.1	0.0001	3.2 (1.6)	4.8 (2.2)	0.0001
Anesthesia of maxilla in an anxious child	3.7	2		2.9 (1.5)	3.4 (2.1)	0.0001
Rubber dam in mandible	1.6	1.8	0.904	1.5 (2)	1.7 (1.7)	0.4
Rubber dam in maxilla	1.6	1.8		1.4 (1.8)	1.7 (1.8)	0.15
Restoration in mandible	1.1	1.3	0.0001	0.6 (0.9)	1.4 (1.4)	0.0001
Restoration in maxilla	1.3	1.5		0.6 (0.9)	1.4 (1.4)	0.0001
Cutting of mandibular crown	1.2	1.6	0.006	1 (1.6)	1.3 (1.7)	0.15
Cutting of maxillary crown	1.3	1.7		1 (1.6)	1.5 (1.8)	0.04
Pulpotomy of mandible	2.4	1.5	0.004	2 (1.5)	2.6 (1.5)	0.0001
Pulpotomy of maxilla	2.5	1.5		2 (1.5)	2.7 (1.5)	0.0001
Pulpectomy of mandible	3	2	0.002	2.2 (1.7)	3.3 (2)	0.0001
Pulpectomy of maxilla	3	2		2.2 (1.8)	3.5 (2)	0.0001
Mandibular tooth extraction	2.8	2.4	0.0001	1.4 (1.5)	3.8 (2.4)	0.0001
Maxillary tooth extraction	2.7	2.3		1.4 (1.6)	3.5 (2.3)	0.0001

## References

[1] Alzahem A.M., van der Molen H.T., Alaujan A.H., Schmidt H.G., Zamakhshary M.H. (2011). Stress amongst dental students: A systematic review.. Eur. J. Dent. Educ..

[2] Locker D. (1996). Work stress, job satisfaction and emotional well-being among Canadian dental assistants.. Community Dent. Oral Epidemiol..

[3] Sugiura G., Shinada K., Kawaguchi Y. (2005). Psychological well-being and perceptions of stress amongst Japanese dental students.. Eur. J. Dent. Educ..

[4] Mahdizadeh M., Kheirkhah F., Vojdani F.H., Noori B.S. (2014). Stress factors in dental students of Babol University.. J Dent Sch.

[5] Rada R.E., Johnson-Leong C. (2004). Stress, burnout, anxiety and depression among dentists.. J. Am. Dent. Assoc..

[6] Pakshir H.R. (2003). Dental education and dentistry system in Iran.. Med. Princ. Pract..

[7] Boran A., Shawaheen M., Khader Y., Amarin Z., Hill Rice V. (2012). Work-related stress among health professionals in northern Jordan.. Occup. Med. (Lond.).

[8] Peres M.A., de Oliveira Latorre Mdo.R., Sheiham A., Peres K.G., Barros F.C., Hernandez P.G., Maas A.M., Romano A.R., Victora C.G. (2005). Social and biological early life influences on severity of dental caries in children aged 6 years.. Community Dent. Oral Epidemiol..

[9] Skaare A.B., Jacobsen I. (2003). Etiological factors related to dental injuries in Norwegians aged 7-18 years.. Dent. Traumatol..

[10] American Academy of Pediatric Dentistry (2013). Guideline on caries-risk assessment and management for infants, children, and adolescents.. Pediatr. Dent..

[11] Ayer W.A. (2005). Psychology and dentistry: Mental health aspects of patient care..

[12] Moulton R. (1955). Oral and dental manifestations of anxiety.. Psychiatry.

[13] Wright G.Z., Kupietzky A. (2014). Behavior management in dentistry for children..

[14] Rønneberg A., Strøm K., Skaare A.B., Willumsen T., Espelid I. (2015). Dentists’ self-perceived stress and difficulties when performing restorative treatment in children.. Eur. Arch. Paediatr. Dent..

[15] Chipchase S.Y., Chapman H.R., Bretherton R. (2017). A study to explore if dentists’ anxiety affects their clinical decision-making.. Br. Dent. J..

[16] DiMatteo M.R., Shugars D.A., Hays R.D. (1993). Occupational stress, life stress and mental health among dentists.. J. Occup. Organ. Psychol..

[17] Gomes H.S., Corrêa-Faria P., Silva T.A., Paiva S.M., Costa P.S., Batista A.C., Costa L.R. (2015). Oral midazolam reduces cortisol levels during local anaesthesia in children: A randomised controlled trial.. Braz. Oral Res..

[18] Myers H.L., Myers L.B. (2004). ‘It’s difficult being a dentist’: Stress and health in the general dental practitioner.. Br. Dent. J..

[19] Morse Z., Dravo U. (2007). Stress levels of dental students at the Fiji School of Medicine.. Eur. J. Dent. Educ..

[20] Polychronopoulou A., Divaris K. (2009). Dental students’ perceived sources of stress: A multi-country study.. J. Dent. Educ..

[21] Davidovich E., Pessov Y., Baniel A., Ram D. (2015). Levels of stress among general practitioners, students and specialists in pediatric dentistry during dental treatment.. J. Clin. Pediatr. Dent..

[22] Aishwarya A., Gurunathan D. (2017). Stress level in dental students performing pedodontic procedure.. J. Adv. Pharm. Educ. Res..

[23] Sofola O.O., Jeboda S.O. (2006). Perceived sources of stress in Nigerian dental students.. Eur. J. Dent. Educ..

[24] Moore R., Brødsgaard I. (2001). Dentists’ perceived stress and its relation to perceptions about anxious patients.. Community Dent. Oral Epidemiol..

[25] Piazza-Waggoner C.A., Cohen L.L., Kohli K., Taylor B.K. (2003). Stress management for dental students performing their first pediatric restorative procedure.. J. Dent. Educ..

[26] Ayers K.M., Thomson W.M., Newton J.T., Rich A.M. (2008). Job stressors of New Zealand dentists and their coping strategies.. Occup. Med. (Lond.).

[27] Simon J.F., Peltier B., Chambers D., Dower J. (1994). Dentists troubled by the administration of anesthetic injections: Long-term stresses and effects.. Quintessence Int..

[28] Gambetta-Tessini K., Mariño R., Morgan M., Evans W., Anderson V. (2013). Stress and health-promoting attributes in Australian, New Zealand, and Chilean dental students.. J. Dent. Educ..

[29] Akbari M., Nejat A., Dastorani S., Rouhani A. (2011). Evaluation of stress level and related factors among students of Mashhad Dental School (Iran) in academic year of 2008-2009.. J Mash Dent Sch.

[30] Chandrasekaran B., Cugati N., Kumaresan R. (2014). Dental students’ perception and anxiety levels during their first local anesthetic injection.. Malays. J. Med. Sci..

[31] Harikiran A.G., Srinagesh J., Nagesh K.S., Sajudeen N. (2012). Perceived sources of stress amongst final year dental under graduate students in a dental teaching institution at Bangalore, India: A cross sectional study.. Indian J. Dent. Res..

